# Protocol for a randomised, placebo-controlled, double-blinded clinical trial on the effect of oestrogen replacement on physical performance to muscle resistance exercise for older women with osteoarthritis of knee joint: the EPOK trial

**DOI:** 10.1186/s12877-023-03828-y

**Published:** 2023-02-18

**Authors:** Tomohiro Mitoma, Jota Maki, Hikaru Ooba, Eriko Eto, Kasumi Takahashi, Tsunemasa Kondo, Tomohiro Ikeda, Yoko Sakamoto, Toshiharu Mitsuhashi, Hisashi Masuyama

**Affiliations:** 1 Department of Obstetrics and Gynecology, Graduate School of Medicine Dentistry and Pharmaceutical Sciences, Okayama University, Okayama, Japan; 2 Department of Obstetrics and Gynecology, Ochiai Hospital, Okayama, Japan; 3 Department of Rehabilitation Medicine, Okayama University, Okayama, Japan; 4 Center for Innovative Clinical Medicine, Okayama University, Okayama, Japan

**Keywords:** Oestrogen replacement therapy, Knee osteoarthritis, Muscle resistance exercise, Sarcopenia, Physical performance

## Abstract

**Background:**

Knee osteoarthritis (KOA) is highly prevalent in older women, and previous studies suggest the involvement of hormonal factors play a role in the pathogenesis of osteoarthritis. KOA causes musculoskeletal impairment, resulting in decreased physical activity, muscle mass, and strength, which leads to sarcopenia and further increases the burden on healthcare systems. Oestrogen replacement therapy (ERT) improves joint pain and muscle performance in early menopausal women. Muscle resistance exercise (MRE) is a non-pharmacological method that preserves the physical functions of patients with KOA. However, data on short-term oestrogen administration combined with MRE in postmenopausal women, especially in those aged > 65 years, are limited. Therefore, this study presents a protocol of a trial aimed to examine the synergistic effect of ERT and MRE on lower-limb physical performance in older women with KOA.

**Methods:**

We will conduct a double-blinded, randomised placebo-controlled trial in 80 Japanese women aged > 65 years living independently with knee pain. The participants will be randomly categorised into two groups: (1) 12-week MRE programme with transdermal oestrogen gel containing 0.54 mg oestradiol per push and (2) 12-week MRE programme with placebo gel. The primary outcome measured using the 30-s chair stand test, and secondary outcomes (body composition, lower-limb muscle strength, physical performance, self-reported measure of knee pain, and quality of life) will be measured at baseline, 3 months, and 12 months, and these outcomes will be analysed based on the intention-to-treat.

**Discussion:**

The EPOK trial is the first study to focus on the efficacy of ERT on MRE among women aged > 65 years with KOA. This trial will provide an effective MRE to prevent KOA-induced lower-limb muscle weakness, confirming the benefit of short-term oestrogen administration.

**Trial registration:**

Japan Registry of Clinical Trials: jRCTs061210062. Registered 17th December 2021, https://jrct.niph.go.jp/en-latest-detail/jRCTs061210062.

## Background

Japan is one of the countries with the highest ageing rate and is rapidly becoming a ‘super-aged’ society; by 2060, 40% of the population or more than 35 million people will be aged ≥ 65 years [1]. Healthy life expectancy is the average number of years an individual is expected to live in a state of self-assessed good or very good health based on current rate of mortality and prevalence of good or very good health [2]. The age difference between healthy and average life expectancies for women and men are 12.3 and 8.8 years, respectively, in Japan [3]. Reduced musculoskeletal function due to joint diseases decreases healthy life expectancy [4, 5]. In a large cohort study conducted in Japan [6], the prevalence rate of knee osteoarthritis was 80.7% and 51.6% among women and men aged ≥ 80 years, respectively, with women having a higher prevalence rate than men [7]. Japan has an estimated 44–48 million potential patients with knee and hip osteoarthritis [8]; the incidence rate is expected to further increase due to the rapidly ageing population.

Knee osteoarthritis (KOA) is the result of wear and tear and progressive loss of the articular cartilage [9]. It causes gradual deformation of the bones around the joint and the joint itself, resulting in joint pain and swelling. Impaired locomotion due to knee joint pain decreases physical activity. In addition, reduced activity leads to the loss of muscle mass and strength, decreased physical fitness, and weight gain, further exacerbating KOA pathology [10]. Decreased lower extremity muscle strength among patients with KOA [11] contributes to sarcopenia, which increases the risk of fall and fracture and the hospitalisation rate, leading to decreased activities of daily living (ADL) and ultimately to increased mortality. Preventive measures are needed before morbid conditions, such as sarcopenia and frailty, and development of and women-centric joint pain interventions are essential for further improvement in healthy life expectancies.

Non-surgical treatment guidelines by the Osteoarthritis Research Society International (OARSI) recommend muscle resistance (MREs), land-based, and water exercises, weight and self-management, and patient education as core treatments [12]. However, frequent interventions [13] and psychological [14] and social factors [15] may hinder sustained exercise motivation. Therefore, MREs need to be short and efficient, and a community system that provides reasons and support systems for continued exercising should be developed.

Oestrogen levels markedly decrease during menopause and are strongly associated with an increase in cardiovascular disease [16] and osteoporosis development [17]. Decreased blood oestrogen levels are also involved in KOA onset, with a significant sex difference and increased incidence in postmenopausal women [18, 19]. Skeletal muscle mass in women continues to gradually decline after peaking in their 20 s [20]; after menopause, the decline rapidly accelerates along with declining muscle strength [21–23]. Moreover, decreasing serum hormones levels, including growth hormone, dihydroepiandrosterone, and insulin-like growth factor-1, are related to a decrease in muscle mass during menopause [24]. Altogether, menopause can accelerate sarcopenia [25]. Oestrogen receptors are present in the articular cartilages [26], tendons, and muscles, and oestrogen helps in maintaining joint homeostasis [27]. Therefore, oestrogen plays a significant role in preventing the onset of osteoarthritis [28, 29] and helps in the development and regeneration of skeletal muscle [30] after menopause. In the Women’s Health Initiative randomised controlled trial (RCT), oestrogen replacement therapy (ERT) in postmenopausal women moderately reduced knee pain frequency and attenuation [31].

The effects of hormone replacement therapy (HRT) on muscle mass and strength in postmenopausal women remain controversial. A recent meta-analysis based on 12 RCTs concluded that HRT without training had no beneficial or detrimental effect on muscle mass, but a significant bias existed in half of the clinical trials [32]. However, a recent clinical trial showed that transdermal oestrogen application with MRE increased the skeletal muscle cross-sectional area compared with that noted with a placebo [33]. A meta-analysis published in 2009 reported that HRT increased the muscle strength in both observational and interventional trials [34]. However, a recent meta-analysis based on nine RCTs of 2,476 postmenopausal women concluded that using HRT without training was not associated with improved muscle strength [35]. Only few studies have investigated the effect of MRE combined with HRT in postmenopausal women [33, 36, 37]. To the best of our knowledge, the synergistic effect of HRT and MRE on muscle strength has not been previously elucidated. Moreover, previous studies [36, 37] on the effect of HRT on skeletal muscles have focused on healthy postmenopausal women in their early 50 s, with only few studies on women in their 70 s and 80 s when muscle weakness begins impacting their lives and few interventional studies on women with joint diseases have been reported.

## Methods/design

### Aim

We designed the EPOK trial to determine the synergistic effect of ERT and MRE on physical performance in the lower limbs of older women with KOA. We hypothesise that oestrogen administration and a muscle resistance exercise programme (MREP) will increase the 30-s chair stand test (CS-30) score, lower extremity strength, and subjective well-being of the participants. If the synergistic effects of oestrogen and the MREP on lower-limb physical function are confirmed, we can administer short-term oestrogen with MREP for KOA and other conditions, such as sarcopenia, wherein physical performance and lower-limb muscle strength are declined.

This protocol complies with the Standard Protocol Items: Recommendations for Interventional Trials (SPIRIT) 2013 guideline [38] for clinical trials and the Consolidated Standards of Reporting Trials 2010 statement [39].

### Study design

**Fig. 1:**
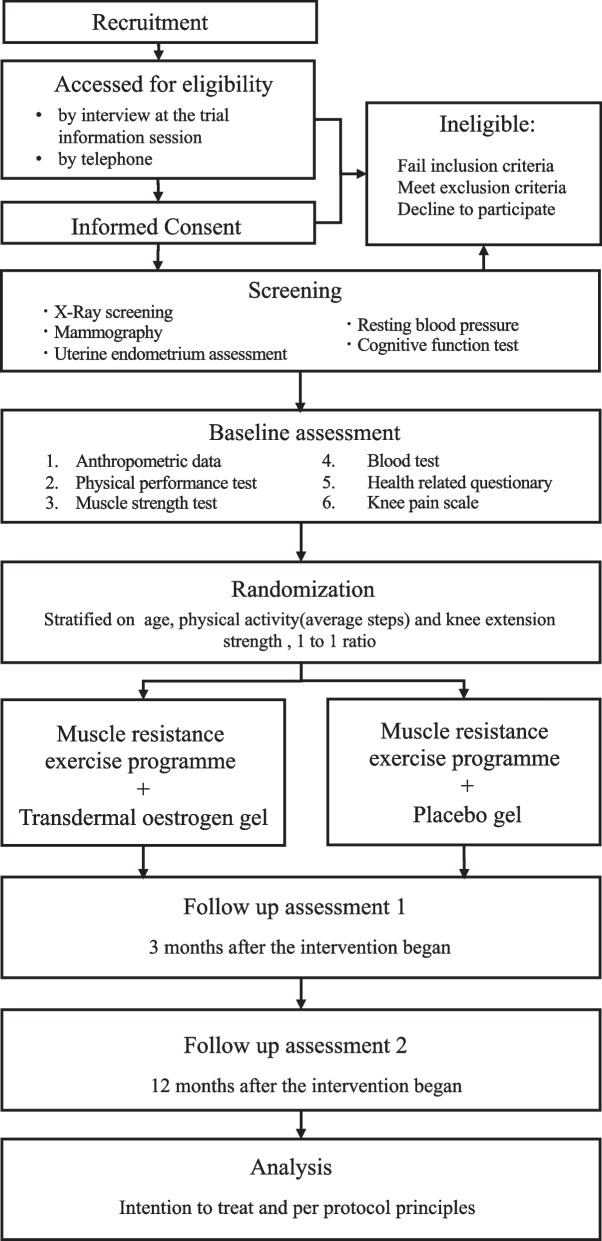
EPOK trial profile. Flow diagram summarizing trial profile through study phases

**Fig. 2:**
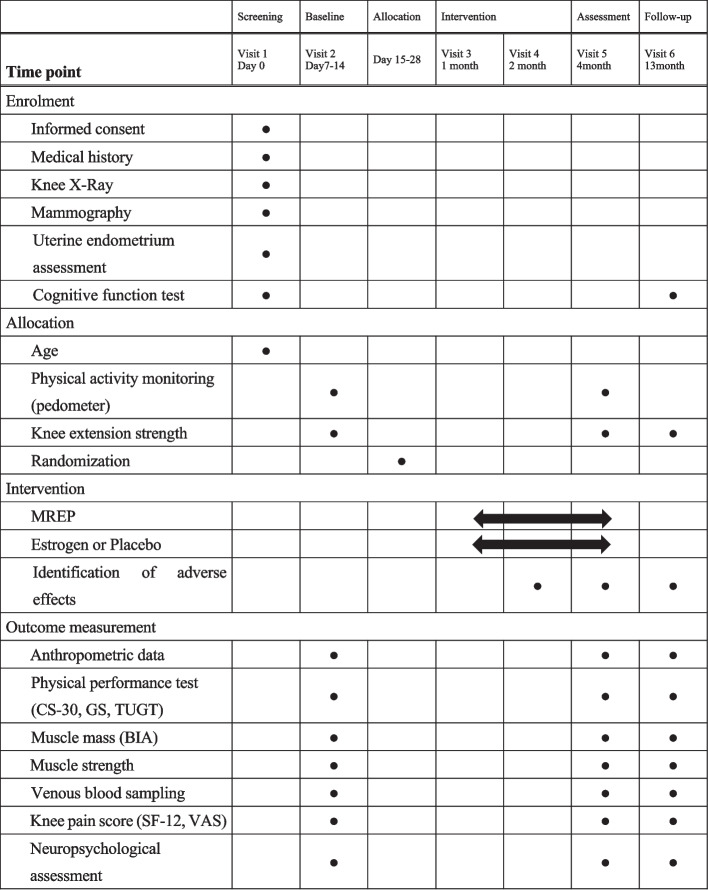
trial design schedule. Study design schedule in accordance with the Standard Protocol Items: Recommendations for Interventional Trials (SPIRIT) Figure

The EPOK trial is a two-arm exploratory double-blinded RCT with a 12-week intervention period, and the outcomes will be measured at baseline, 3 months, and 12 months. Figure 1 outlines the RCT phases. Eighty participants will be identified and randomly categorised into two groups: (1) MREP with transdermal oestrogen gel and (2) MREP with placebo gel. Eligibility will be assessed during screening. The Okayama University Hospital and Ochiai Hospital will conduct this trial. We have organised the EPOK trial team, including the Maniwa City Community General Support Center, which will assist with recruitment via telephone, mail, local newspapers, and public health seminars. The non-profit organisation (NPO) of the agri-garden project will assist with MREP coaching. Figure 2 outlines the trial design schedule following the SPIRIT guidelines [38].

### Participants

Eighty women will be recruited from the community in Maniwa City, Okayama, Japan. The EPOK trial eligibility criteria include:

- 1)Women aged between 65 and 90 years when obtaining their consent.
- 2)Participants with KOA with knee pain lasting ≥ 3 months.
- 3)Those able to ambulate independently.

The exclusion criteria include [40]:

1. Disease diagnosed within the previous 2 years, including myocardial infarction, severe aortic stenosis, acute pericarditis, acute myocarditis, aneurysm, severe angina, clinically significant valvular disease, uncontrolled dysrhythmia, thrombophlebitis, pulmonary embolism, claudication, cerebral palsy, home oxygen therapy, psychotic illness, or depression, or conditions causing difficulty in exercising.
2. Diagnosed with endometrial, ovarian, peritoneal, cervical, or breast cancers within the previous 5 years.
3. History of upper or lower-limb bone fractures within the previous 6 months or 2 years, respectively.
4. Bedridden condition for > 1 week within the previous 2 months, or for > 2 weeks within the previous 6 months.
5. Inadequate intake.
6. Renal impairment.
7. Severe liver function disorder.
8. Resting systolic pressure > 200 mmHg or resting diastolic pressure > 100 mmHg.
9. Cognitive impairment.

### Study procedures

#### Recruitment

The potential participants will be recruited by medical practitioners and local social life support coordinators. Flyers and posters will be mailed to approximately 1000 seniors in 100 local senior groups, which exist as databases in the community support centre. In addition, the trial team will approach the senior worker group via telephone and mail the flyers.

### Screening and consent

Interested volunteers will be invited to attend an information session held by the chief investigator (CI) in each local district or senior group community. They will undergo screening via interviews using the medical history and exclusion criteria checklist. They can be screened via telephone if they cannot attend the interviews. If no obvious exclusion criteria exist, the volunteers will undergo a medical assessment as potential participants. All potential participants will provide written informed consent after the aim, treatment plan, anticipated benefit, and potential hazards of this trial are explained to them by the CI or sub-investigator. Furthermore, they will have the opportunity to ask questions during this time.

### Medical assessment

The potential participants will undergo a medical assessment after providing written informed consent, which will include medical history, clinical examination (knee, breast, and endometrial assessments), and cognitive assessment, to meet the inclusion and exclusion criteria. Medical history assessment will detail the current medical condition of the participant, including medication usage, family, past medical, and social (occupation and activity participation) histories, substance use/abuse (alcohol and smoking), and allergies. Knee radiographs and interviews regarding knee pain will be used for KOA clinical examination. If participants have already been diagnosed with KOA [41], they can skip the knee radiography. Participants will be interviewed regarding the location, duration, and pain intensity on a 0–10 visual analogue scale (VAS). Knee radiographs with Kellgren and Lawrence classification [42] of at least grade 1 (doubtful narrowing of the joint space with possible osteophyte formation) will be considered as positive. Patients with spontaneous knee pain in the previous three months and positive knee radiographs will be eligible for our trial. Breast and uterine endometrial examinations will be performed to minimise the complications of ERT [43], including mammography and ultrasonography, to exclude breast and gynaecological cancers. If a suspicious lesion is identified on breast examination or if endometrial thickness is > 5 mm [44], the participant will be excluded from the trial and referred for further examination. Resting blood pressure will be measured during the screening; participants with systolic pressure > 200 mmHg or diastolic pressure > 100 mm Hg will be excluded from the trial. Cognitive function tests will be performed using the Revised Hasegawa Dementia Scale. Participants scoring ≤ 20 on a 30-point scale, indicating a suspicion of dementia [45], will be excluded from the trial.

### Randomisation, allocation, and blinding procedure

Each participant will be enrolled in this trial by investigators after the medical assessment and matching the inclusion and exclusion criteria. The investigator will provide each participant with an identification code according to the case enrolment order and fill out a registration sheet, which includes the date of obtaining consent, any other information necessary to match the participant with the corresponding identification code, and the factors used for stratified randomisation.

The participants will be randomly categorised into two groups by an allocator who will not have contact with them throughout the trial: (1) MREP with transdermal oestrogen gel and (2) MREP with placebo gel. The participants will be stratified according to age (older: 75–89 years, younger: 65–74 years), physical activity (the intermediate steps taken in a week measured by a pedometer, higher ≥ 5000 steps per day, lower < 5000 steps per day), and knee extension strength (higher ≥ 18 kgf, lower < 18 kgf). The randomisation schedule will be computer-generated using the randMS® mobile application [46] (sold by Shinji Maeda, created using Filemaker® and Filemaker Pro Advanced®, ©2019 kazenoan) with a mixture of two or more block sizes. The allocator will send an email with the trial group allocation result and participant identification codes to an unblinded pharmacist who will fill the placebo gel into the supplement bottles and label them according to the group allocation. In the unlikely event of an adverse symptom requiring further medical investigation, the gel allocation details can be accessed by the investigator, and the patient will be excluded from the trial.

### Sample size and power analysis

The sample size calculations were based on previously published data on increased lower extremity strength with MRE and ERT [47]. The rate of increase in the muscle strength during ERT is 5%–17% [34]. Positive correlations (*r* = 0.75) have been reported regarding the relevance of CS-30 scores on knee extension strength [47]. The average CS-30 score in patients with KOA is 9.2 repetitions [48]. Assuming that ERT can be expected to increase the CS-30 score by 13%, this sample size allows the detection of 1.2 times the primary outcome improvement (increased CS-30 score), in addition to an increase of 2.68 repetitions (1.90–3.47 repetitions, *p* < 0.001; I2 = 0.50%) due to the MREP alone [49]. The required sample size is 36 persons per group, calculated with an 80% one-sided test setting power, 5% alpha, and a 0.6 effect size (Cohen's d). The target size is set to 40 per group to account for a dropout rate of approximately 10%. Reports have shown that ERT has a low-to-moderate effect on lower extremity strength [34, 49, 50] and that ERT has improved knee pain and joint function in patients with KOA [29, 31]; therefore, we assume that ERT will have a slightly larger-than-moderate effect.

### Statistical analysis

The MREP participants will be assigned to either the oestrogen or placebo group. Data will be aggregated for the assigned analysis participants at baseline, 3 months, and 12 months after the intervention. The target population will be the full analysis set, defined as all the randomised participants who have started their trial treatments.

The statistical analysis will be based on the intention-to-treat and per-protocol principles. The participants’ demographic background and baseline characteristics will be described as appropriate, with data presented as mean and standard deviation for continuous variables that appear to be approximately normally distributed, as medians and interquartile ranges for other continuous variables, and as counts and percentages for categorical variables. Linear regression analysis will be performed for a statistical comparison of the primary and secondary outcomes between the oestrogen and placebo groups. The relationship between the intervention and endpoints will be calculated as a regression coefficient with a 95% confidence interval. The covariate distribution is expected to be consistent across the groups due to randomisation. Therefore, only the presence or absence of intervention will be used as an explanatory variable for the linear regression analysis. However, the variable distribution will be biased by chance due to allocation; thus, the variable is added to the explanatory variables and an adjustment is made. We will adopt a complete case analysis as the primary analysis if the proportions of missing data are < 5%. Multiple imputations will be conducted if imputation of > 5% is required for the amount of missing data for an outcome, and the method will be reported.

The statistician, who is blinded to the allocation, will perform the analysis at the Department of Obstetrics and Gynaecology, Graduate School of Medicine, Dentistry, and Pharmaceutical Sciences, Okayama University. The validity of the analysis will be verified by the Centre for Innovative Clinical Medicine and Department of Epidemiology of the Okayama University as a third-party institution.

### Data collection and management

The data collection schedule is illustrated in Fig. 2. Data will be obtained from the Ochiai Hospital. The investigator will collect the potential participants’ information (name, home address, date of birth, and contact number) during the first visit to the hospital after recruitment. The participants will be assigned a participant identification code after trial enrolment and their data will be managed according to it. A list of the participants’ personal information, participant identification codes, consent forms, case report forms, and other documents or records necessary to ensure data reliability will be securely stored at the Ochiai Hospital in a locked cabinet, accessible only to the trial team members. All the collected data will be entered into a password-protected Excel 2019 sheet (Microsoft Corporation, Redmond, Washington, USA). These data will remain for 5 years after the end of the trial, and will thereafter be shredded and disposed of with great care to protect the participants’ personal information.

### Monitoring

This trial will be monitored by a person assigned by the CI in accordance with the Standard Operating Procedures for Monitoring at Medical Institutions. Onsite monitoring shall be conducted by visiting the medical institution for direct trial implementation system confirmation and source document inspection. Among the 80 patients, 10 will be monitored. However, the sampling rate will be increased by 30% of the total number of patients if a problem arises in even one patient. Monitoring will include verifying the clinical trial progress and confirming whether all the required procedural documents are present at the research office through electronic communication. Additionally, the monitor will record the information, materials, and reports obtained throughout the trial and store them securely. Monitoring reports will be made available to the Okayama University Certified Review Board.

### Interventions

All the participants will receive instructions regarding the MREP and transdermal gel. They will be provided with educational information about the advantages of lower-limb exercises in KOA and the importance of adherence to the MREP. Analgesic medication use will be allowed, including nonsteroidal anti-inflammatory drugs as transdermal gels for the knee joint. All participants can continue their normal daily activities, such as walking and stretching. However, they will be asked to avoid new invasive knee joint treatments, including surgery and fluid injection.

### Muscle resistance exercise programme

The MREP will implement the same content for both the intervention groups, with one training session per week to be held in a group and two sessions per week at home. Twelve group sessions will be held at the community centre in each district where an instructor will be present to ensure training quality and provide guidance. The Maniwa Agri-garden-project NPO provides the MREP instructors with a group session. The exercise instructors will have a 2-h lesson provided by an experienced physiotherapist from the Okayama University Hospital and will receive certified coaching insurance from the CI. Each group session will last 90 min, including a 15-min break.

The group MREP session will comprise an evidence-based programme designed by a physiotherapist at the Okayama University Hospital. The MREP is a multi-component exercise programme with a beneficial effect on the physical functions of patients with KOA, consistent with the current standard of care and clinical practice guidelines [51–53]. It includes a dynamic warm-up with range of motion stretches (approximately 10 min), MRE (approximately 25 min), balance training [54] (approximately 10 min), cool-down with stretches (approximately 5 min), and a coordinated dual-task section [55] (approximately 25 min). The MRE was designed based on the OARSI guideline [56, 57], a structured land-based exercise programme focusing on the four major lower-limb muscle groups (quadriceps, calf muscle, hamstrings, and gluteal muscles), and abdominal and back muscles in patients with KOA. The MRE section consists of chair-based and standing exercises, including knee extension, knee lift exercises from sitting and standing positions, heel raise, squats, and chair standing exercises. Each movement will take 6–8 s, with the participants counting the seconds out loud. Each exercise will comprise two sets of 8–10 repetitions with gradually increasing muscle load [54] and number of repetitions based on the participant's perceived exertion. Weeks 1–4 will have no weight load, and 1.0 kg ankle weights will be used to increase the muscle load from week 5. The exercise instructor will supervise the participants by providing verbal feedback to ensure that the target muscle groups obtain sufficient tension.

The home training session will be scheduled on two non-consecutive days per week. It will comprise a 25-min MRE, identical to that of the group session. Each participant will receive a pamphlet regarding the home training content and a calendar form pictorially depicting the MRE. Participants will have to ‘circle’ the days on which they have performed the MRE at home to track whether they have performed the exercise. Furthermore, exercise quality will be evaluated by writing a numerical self-assessment from 1 to 3 on the calendar (1: performed less than half, 2: performed half, but not all, 3: completed all). The instructor will check the calendar contents during the group session every 4 weeks and provide guidance to those who need instruction.

### Transdermal oestrogen gel

During the intervention, l’ oestrogel 0.06%®　(gel containing 0.06% oestrogen; Fuji Pharma, Tokyo, Japan) (Standard Commodity Classification Number of Japan 872,473, approval number: 21800AMY10135, YJ code: 2473700M2026) [58] or placebo gel will be used. The transdermal oestrogen gel used in this trial is a clear, colourless gel containing 0.54 mg oestradiol per push (0.9 g), with an internal volume of 80 g per bottle [58]. The gel-type application prescribed by the CI will be applied once daily at a fixed time before beginning the MREP; one push should be applied as widely and thinly as possible on the anterior surface of the thigh with the palm. To ensure blinding, an unblinded pharmacist, who does not prescribe and treat subjects directly, will be set up to apply the bottle sticker. The bottles containing the oestrogen or placebo gel will be designed to ensure an accurate and uniform quantity dispensation with each push. The bottles will be packaged and distributed with a sticker bearing the participant identification codes. The bottles will be stored indoors at 1–30 °C to avoid heat, moisture, and sunlight. The placebo gels will be composed of ingredients similar to the base drug substance of the oestrogen gel, with comparable colouration, odour, and texture. The main ingredients of the base are ethanol, which is also used in the actual drug xanthan gum [59] as a viscosifying agent, and glycerol [60] as a moisturising ingredient. Both substances have already been proven to have no effect on the human body and are used in commercial moisturisers [59, 60]. The placebo drug will be prepared by an unblinded pharmacist at the Ochiai Hospital and injected into bottles, which will appear the same as the actual drug bottles, ensuring the quality of external blinding. The bottles will be checked during outpatient visits conducted one month after starting trial drug administration to check for side effects, and the amount used will be determined based on the amount remaining in each bottle.

### Outcome assessment

Outcome assessment will include baseline measurements of participants' anthropometric data and outcomes. At baseline, a physiotherapist will measure the height, weight, and upper arm, thigh, calf, abdominal, and gluteal circumferences and calculate the body mass index (BMI). The resting blood pressure and heart rate will be measured simultaneously. Outcome examinations will be performed at baseline before allocation, and 3 and 12 months after intervention initiation (Fig. 1). Outcomes will be measured by physical therapists with at least 5 years of clinical experience.

The primary outcome will be the 3-month and 12-month changes from the baseline physical performance according to the CS-30 score. The CS-30 devised by Jones is highly reproducible and has been shown to correlate with the quadriceps muscle strength and the ability to balance, walk, and perform ADL [61]. Since KOA-related knee pain reduces the ability to perform vertical movements, CS-30 is also reliable for functional assessment of inactivity due to KOA [62]. CS-30 uses an armless folding chair with a seat height of 40 cm. The participants will be seated in the middle of the chair with their back straight, feet shoulder-width apart, and arms crossed at the chest, and will be encouraged to complete as many full stands as possible within 30 s [61]. They will be instructed to sit fully between each stand. The measurer will silently count the correct stand completions after letting the participant practice once or twice.

The secondary outcomes include:

1. Change in the knee pain score assessed using the VAS and 12-item Short form survey (SF-12).

VAS will be used to assess knee joint pain before and after the intervention. The pain intensity will be indicated by a 10 cm black line (the left end for ‘no pain’ and right end for ‘the greatest pain imaginable’) on the questionnaire, and the participant will be asked to draw a line to indicate the level of her current pain. Health-related quality of life as pain in daily life will be assessed using the SF-12, which was validated as a shorter alternative to the SF-36 [63].

2) Change in physical function according to the gait speed and timed up and go test (TUGT)

The participants will be asked to walk along an 11 m straight line as quickly as possible, excluding the first and last 3 m. The 5-m walking time will be measured twice and the better result will be considered. The gait speed will be calculated as distance (m)/time (s). The TUGT measures the time taken to walk, after sitting in a chair, and turning around at a cone 3 m away to walk back and sit in the chair again. The TUGT is widely used in geriatric settings to assess lower-limb strength and fall risk [64].

3) Change in muscle strength according to the hand-grip strength and knee extension strength (KES)

We will use a digital hand-grip dynamometer (Takei-kiki Corp., T.K.K5401) to measure the hand-grip strength and a hand-held dynamometer (HHD) with a fixing belt (Anima Corp., Mu-Tas F-2) to measure the KES. The participants will be instructed to maintain an upright posture, with the hip and knee joints bent at 90° in the end-sitting position. The HHD pad will be placed on the participant's shin, with the pad secured to the chair with a fixing belt. The operator will provide light support to prevent the pad from shifting during measurement [65]. The participant will gradually increase force to maximise knee extension for 5 s and hold the knee joint for 2 s at the point of maximum extension. Each strength measurement will be performed on the left and right sides and the higher value will be considered. The maximum force will be recorded in kilograms (kg) for the hand-grip strength and in kilogram-force (kgf) for the KES. The KES with HHD will be normalised to their mass (kgf/kg). An average KES of 18.8 ± 3.2 kgf in healthy women in their 80 s [66] and a positive correlation (r = 0.385) between KES and sit-ups [67] have been previously reported.

4) Changes in muscle mass assessed by bioelectrical impedance analysis (BIA)

To assess muscle mass, body composition will be obtained by BIA measurements using the Tanita MC-780AN and Tanita Zaritz BM-220 (Tanita Corp., Tokyo, Japan) instruments. Appendicular skeletal mass (ASM, kg), BMI (kg/m^2^), skeletal muscle index (ASM/m^2^), total lean mass (TLM, kg), Body muscle index (TLM/weight × 100), and fat-% will be the body composition variables.

5) Blood sampling

The levels of albumin, creatinine, bilirubin, alanine aminotransferase (ALT), aspartate aminotransferase (AST), gamma-glutamyltransferase, C-reactive protein, low-density lipoprotein, high-density lipoprotein, total cholesterol, triglyceride, oestradiol, insulin-like growth factor-1, calcium, 25-hydroxy vitamin D, d-dimer, fasting glucose, haemoglobin A1c, haemoglobin, and haematocrit and the white blood cell (WBC) and platelet counts will be assessed in all participants at baseline and after the intervention.

6) Neuropsychological assessment

A verbal fluency test [68] will be performed for all participants at baseline and after the intervention.

### Adverse events

The drug safety tests in this trial include liver and renal function tests, serum cholesterol levels, mammography, and gynaecological examination. Collection and examination of these parameters and all blood samples will be conducted at the Ochiai Hospital, Okayama, Japan. Participants will have 24-h access to call and visit the emergency department of Ochiai Hospital if any adverse event is suspected. A standardised protocol for potential adverse events will be available at the Ochiai Hospital with the CI’s contact number and a manual for examination and testing. All the adverse events will be recorded and monitored until the end of the trial. All significant and severe adverse events will be recorded in the clinical notes and written in a case report form, which includes the adverse event details, such as the timing of onset and resolution, extent, treatment, outcome, and severity assessment. Significant and severe events will be reported by the Okayama University Clinical Research Board (CRB) using the application form on the internet site. If participants have significant or severe adverse events, the CRB or CI will determine whether the participants can continue with or withdraw from the trial and inform them accordingly. These participants will be referred and treated for each event depending on the symptoms and severity, even if they are withdrawn from the trial.

The participants may report minor adverse events [58, 69] including:

- Reproductive organ: irregular vaginal bleeding, increased vaginal discharge, vulval irritation or itching, increased leiomyoma size, endometriosis recurrence
- Breast: tender breasts
- Skin at site of gel application: redness, irritation, itching, pigmentation, hives
- Nervous system: headache, migraine, weakness, dizziness, depression, nervousness, rapid changes in mood, difficulty sleeping
- Cardiovascular: palpitation, rise in blood pressure
- Gastrointestinal: abdominal distension, nausea, abdominal cramps, vomiting, heartburn
- Liver function: increased liver enzymes (AST, ALT, alkaline phosphatase)
- Blood: decreased WBCs, anaemia
- Musculoskeletal: back pain, lower limb, ankle, or finger swelling, arthritis
- Others: Increased triglycerides, fatigue, tinnitus, nasal bleeding, weight gain

Significant adverse events [59, 70] include heavy vaginal bleeding, jaundice, heavy abdominal distension, and oliguria.

Severe adverse events [58, 69] include:

- 1) Any sign of anaphylactic shock: Skin reactions, including hives and itching, and flushed or pale skin, low blood pressure, airway constriction, swollen tongue or throat (which can cause wheezing and trouble breathing), weak and rapid pulse, sudden nausea, vomiting, diarrhoea, or rapid change of consciousness.
- 2) Any sign of thrombosis formation: pain and oedema in the lower extremities, chest pain, sudden shortness of breath, acute visual impairment.

### Amendment

If the authors wish to make any substantial amendments, changes, or revisions to the research protocol or consent, documents will be approved in advance by the Accredited Clinical Research Review Board. When the following minor changes are made to the implementation plan, the Okayama University Certified Review Board will be notified of the details within 10 days of the changes:

- 1) Notification of changes regarding the implementation plan.
- 2) Revision of the implementation plan.
- 3) Notification of the review results by the Okayama University Certified Review Board.

### Dissemination plan

The results of this trial will be submitted for publication in a journal to disseminate information to the health network. When the trial results are public, participants’ names, dates of birth, and other information that can directly identify them shall not be included. The published results will be limited to those that underwent statistical processing. The participants will receive a copy of the published article by mail.

## Discussion

Musculoskeletal disorders, including osteoarthritis [11] and osteoporosis [17], are more common in postmenopausal women, and the decline in muscle strength and physical function due to these effects can lead to a ‘negative spiral’ of falls, fractures, and sarcopenia development [7]. Therefore, preventive measures are required for these women. The EPOK trial is the first study to focus on the synergistic effect of oestrogen and MRE on KOA in women older than 65 years. If this trial's successful implementation proves the effectiveness of ERT in improving physical function in older women, it could lead to effective short-term interventions during MRE for joint diseases and muscle weakness, such as sarcopenia.

One of the strengths of this EPOK trial is that it is a placebo-controlled, double-blind RCT that adequately measures oestrogen effects on the musculoskeletal system using primary and secondary outcomes. However, we will not perform muscle or cartilage biopsy to investigate the oestrogen effect using immunohistochemical methods because of the high invasiveness and burden of frequent hospital visits for the participants.

In this trial, two interventions will be implemented to observe their effects on the musculoskeletal system, particularly in the lower extremities. Based on previous evidence, the MREP will be a multi-component programme. The weekly coaching will ensure exercise quality, and home training will allow the participants to monitor the amount of exercise done using photo handouts and filling-in exercise assessments. Transdermal oestrogen administration reduces the side effects on hepatic metabolism such as thrombosis and increased triglyceride levels. The gel formulations will be simple to administer, and the bottles will be designed to ensure consistent and accurate dosage. The 12-week intervention period will be adequate to ensure exercise continuity and measure the effects of oestrogen administration. The interventions are practical due to close collaboration with municipal healthcare services, including local governments and NPOs. Thus, these two interventions can be easily and accurately implemented.

Improving exercise adherence requires careful adjustments of the intervention frequency [13] and location [71]. Moreover, to sustain exercise and group activities after the trial, we must consider each participant's psychological [14] and social background [15]. The EPOK trial is a collaborative study between university hospitals, local governments, and NPOs. Once the framework for cooperation between local governments and organisations is implemented in this study, the participation of older women in social activities will be promoted following discharge from the study.

### Study status

Recruitment is ongoing at time of manuscript submission.

## Data Availability

The datasets used and/or analysed during the current study are available from the corresponding author on reasonable request.

## Abbreviations

KOA: Knee osteoarthritis
ADL: Activities of daily living
OARSI: Osteoarthritis Research Society International
MRE: Muscle resistance exercise
ERT: Oestrogen replacement therapy
RCT: Randomised controlled trial
MREP: Muscle resistance exercise programme
CS-30: 30-Second chair stand test
NPO: Non-profit organisation
CI: Chief investigator
VAS: Visual analogue scale
BMI: Body mass index
SF-12: Short form survey
TUGT: Timed up and go test
KES: Knee extension strength
HHD: Hand-held dynamometer
BIA: Bioelectrical impedance analysis
ASM: Appendicular skeletal mass
ALT: Alanine aminotransferase
AST: Aspartate aminotransferase
WBC: White blood cell
CRB: Clinical Research Board
